# Teaching controversial socio-scientific issues in online exhibits of science museums: Covid-19 on the scene

**DOI:** 10.1186/s43031-022-00069-8

**Published:** 2023-01-02

**Authors:** Carolina Sotério, Adriele Ribeiro dos Santos Lamim, Salete Linhares Queiroz

**Affiliations:** São Carlos Institute of Chemistry, São Carlos, University of São Paulo, São Paulo, Brazil

**Keywords:** Covid-19, Socio-scientific controversies, Museums, Online education

## Abstract

The Covid-19 pandemic has sparked an unprecedented public debate over socio-scientific controversies, particularly regarding vaccination and social distancing measures. Despite the potential of such subjects for developing critical thinking and a sense of citizenship, the theme of controversies is still incipient in science museums. This documentary study investigates the way three science institutions have proposed online exhibits on Covid-19 on Google Arts & Culture platform and checks their potential for favoring teaching on controversial science topics. Google Arts & Culture platform was searched for Covid-19-related keywords and the filtering of the results was based on focus and organizing institutions. Three exhibits were detected, whose analysis was submitted to an inter-rater agreement (Cohen’s kappa). The results revealed the predominance of social and economic aspects that can strongly favor more scientifically progressive views of both science literacy and a socially undistorted science. On the other hand, the superficiality of political discussions on science topics, a lack of naturalization of the controversial discussions, and an excessive use of textual content were identified, thus revealing some initiatives have not explored the interactivity, multimediality, and the way dilemmas that mark the trajectory of science museums extend to online exhibits. From this perspective, we point out paths for teaching and learning socio-scientific controversies in museums.

## Introduction

The educational process aims at human development and provides access to the civilizational heritage, which consists of knowledge, skills, and products that help to overcome the limits imposed by nature and promote the scientific and cultural evolution of humanity (Rodrigues, 2001). In this sense, museums are important educational spaces, since they enable contact with knowledge and products from previous generations through the exhibition of their collections and objects. However, this is not the only aspect that makes those institutions places of teaching and learning.

Museums are currently understood as environments that promote non-formal education (Marandino, 2008; Taylor & Neill, 2008), i.e., an educational process with specific objectives planned by an institution that is not part of the formal education system, which is comprised of compulsory and higher education establishments (Zabala & Roura Galtés, 2006). Non-formal places of instruction do not replace schools, but are associated with them, contextualizing the contents worked in classrooms (Santos et al., 2017).

Museums are conceived as an important field within the non-formal education sector (Pastor Homs, 2009), since collections, exhibitions, buildings, websites, lectures, publications, and their teaching materials constitute a source of resources that provide countless possibilities for learning. The educational basis supports museological activities; it is present from the construction of an exhibition script, arrangement of objects, and writing of explanatory texts to the contact of an instructor with visitors (Domínguez, 2009), going beyond a mere presentation of content, thus awakening creativity, questioning, critical thinking, reflection, and (re)construction of knowledge, configured in an educational act (Santos, 2001).

Besides the aforementioned tasks, science museums perform various social functions, teaching and communicating science, promoting science literacy, investigating and disseminating the culture of study fields, preserving the environment and promoting awareness for its preservation, training specialists, and arousing public interest in scientific topics (Delicado, 2004). Furthermore, they present the developments and discoveries of national science, encourage public engagement in decisions on science and technology spheres, and debate controversial science topics (Delicado, 2007), defined as.

(...) those topics that have connections to science and inspire complex decision making about issues of societal or personal relevance. This includes societal controversies (…) such as genetic cloning and global climate change, which have been defined as issues that are ‘based on science concepts or problems, controversial in nature, discussed in public outlets, and frequently subject to political and ethical influences’ (…) We also include personal controversies within our conception of controversial science topics, which we define as science-related issues individuals face in their everyday lives, as they decide such things as what to eat, what kind of medical treatment to seek, or whether to engage in sexual behavior (Brotman et al., 2011, p. 89).

Such topics have been especially observed during the Covid-19 pandemic, which, in addition to a health crisis, has revealed several other associated problems, such as racism, xenophobia, unemployment, and hunger (Blustein et al., 2020; Elias et al., 2021; Sinha, 2021), as well as the emergence of an infodemic, i.e., the circulation of a massive volume of information (not necessarily accurate) facilitated mainly by social media. The phenomenon has impacted public debates on social, political, and scientific issues, thus bringing controversial topics such as vaccination, social distancing, and science denialist movements to the spotlight. According to the World Health Organization (WHO), a joint effort by researchers, governments, and other community members is necessary for combatting the crisis effects, since the dissemination of information with no scientific evidence can represent a threat to life (World Health Organization, 2020).

The Covid-19 communication crisis has also affected other media outlets. Podcasts, intrinsically connected with the mobility of their listeners, were initially impacted by the lockdowns and needed to be re-signified. As a result, the consumption profile on Spotify – one of the biggest audio streamers – has changed, indicating an increase in the listener’s preference for news content (Spotify, 2020). On the other hand, the same platform hosted podcasts that spread misinformation about Covid-19, leading to a global protest and concern about the content accuracy from streamers that reach large audiences (Sisario, 2022).

In the United States (US), the time people spend watching TV significantly increased in 2020 compared to the previous year, indicating a viewer’s preference for traditional media as a source of accurate information (Casero-Ripollés, 2021; Nelson, 2020; Nielsen, 2020). However, despite holding people’s attention, not all media were really addressing topics of public interest – an analysis of the websites of 50 US state broadcasting associations revealed a prioritization of industry practices over community-related issues (Blaney & Hunt, 2021).

Despite its importance, discussing controversial science topics has been little explored by museology. Most of the time, museums present knowledge as something finished and unquestionable, through either written texts, or even interactive devices, thus leaving little space for debate, questioning, and the unknown (Arnold, 1996; Butler, 1992; Delicado, 2007; Huddleston & Kerr, 2015; Pedretti et al., 2018).

This study aims to detect the elements present in three online museum exhibits on Covid-19, namely Coronacene – Thoughts in Times of Pandemic (Museum of Tomorrow, 2021), COVID-19 – Mass manufacturing a vaccine (Museum of Engineering Innovation, 2020), and Field in focus: predicting pandemics (Smithsonian National Zoological Park, 2020) that can potentially enable teaching on controversial science topics. The analysis of the elements used (or not) by the exhibits to address such themes can help teachers, curators, and museum instructors reflect on both construction of this type of exhibition and its educational potential.

The following research questions have been raised:

1. *To what extent do virtual exhibitions on Covid-19 organized by science, technology, engineering, and mathematics (STEM) institutions include elements that address controversial aspects of the pandemic?*
2. *Does the way in which such elements are present in the visual, audio, and textual contents of the exhibits created by STEM institutions and published on Google Arts & Culture contribute to the teaching of controversial socio-scientific topics?*

Virtual exhibits of science museums are also held on platforms other than Google Arts & Culture (e.g., self-owned websites and social media accounts). However, the present investigation prioritized a consolidated platform that gathers and standardizes online exhibitions for comparing their contents.

## Methods

**Fig. 1:**
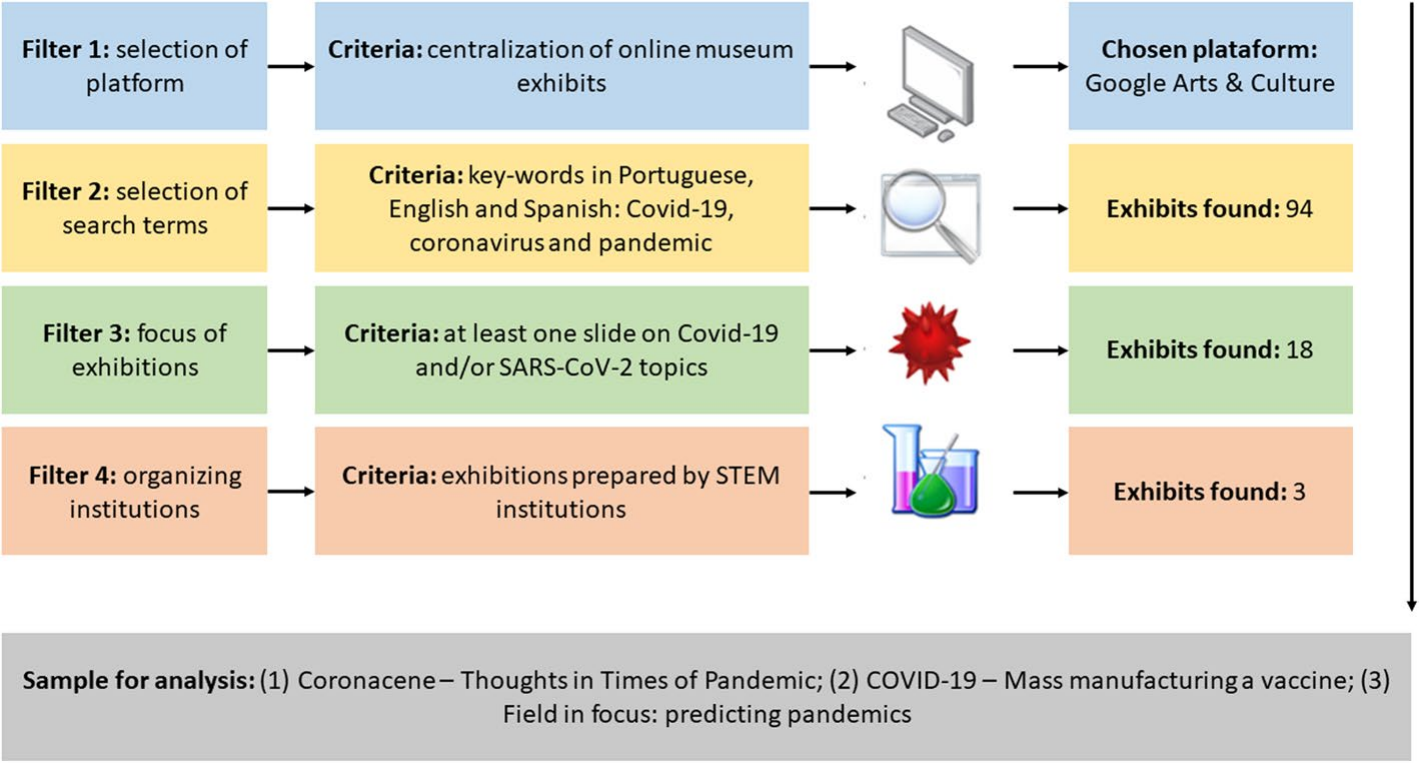
Execution steps of the survey on online exhibits on Covid-19

This research is exclusively documentary, i.e., devoted to the mapping of a field of knowledge towards discussions on both aspects privileged to the detriment of others and possible paths from the results (Romanowski & Ens, 2006). The three museum online exhibits on Covid-19 are presented from the perspective of teaching controversial science topics. An examination of online exhibits was conducted by the authors and considered exhibitions published between March 11, 2020, which dates the WHO pandemic status, and October 1, 2021, the date of this analysis, whose execution steps are shown in Fig. 1.

Due to the Covid-19 pandemic, virtualizations began to play a leading role in socio-cultural relationships, among which museum visits stand out (Lerario, 2021), and lockdown measures adopted as public health policies in many countries led some museums to use online platforms for connecting with their audiences in the midst of the health crisis (Vayanou et al., 2020). According to the International Council of Museums, ICOM, at least 50% of museum institutions sought digital communication channels (e.g., social media, live streams, and online educational actions) during that period (International Council of Museums, 2020).

In this scenario, Google Arts & Culture is a free platform that has stood out for its power for mapping cultural actions at an international level and simulating virtual tours, featuring a range of audiovisual tools such as 360° videos and street view navigation (filter 1). Despite the efforts to maintain activities online, several institutions do not have the necessary infrastructure, and 40% of the world population still do not have access to the Internet (Kemp, 2021).

Keywords “pandemic”, “Covid-19”, and “coronavirus” – in Portuguese, Spanish, and English – were inserted in the platform’s search tool for filtering museum online exhibits on the subject of study, thus resulting in 94 unique “stories” (filter 2). Exhibits that focused on the Covid-19 pandemic, of which some addressed the disease and/or the SARS-CoV-2 virus, were then identified, resulting in 18 initiatives that addressed topics such as abrupt changes in everyday life, personal thoughts and feelings, face masks, vaccines, wildlife, frontline workers, and sociopolitical issues and materialized them in audios, videos, illustrations, comics, photographs, paintings, billboards, poems, and other textual types (filter 3). Finally, the exhibits prepared by STEM institutions were selected (filter 4), thus leading to three productions, namely Coronacene – thoughts in times of pandemic, COVID-19 – mass manufacturing a vaccine, and Field in focus: predicting pandemics, detailed below. Such exhibits are dynamic, i.e., comprised of slides that move up and down across the screen, according to the mouse cursor

### Elements common to the teaching of controversial science topics

From a literature review, Huddleston and Kerr (2015) argued on the pertinence of teaching controversial science topics based on (a) *product-based justifications*, emphasizing (i) the relevance of discussing social, political, economic, and moral issues, important to the student life, and (ii) compensating for the one-sided and confusing presentation of subjects made by the media. In contrast, (b) *process-based justifications* include reasons that are:

(i) *Subject-related* (e.g., understanding that controversy is not to be feared, but part of life in a democracy, the ability to discuss contentious issues in civil and productive ways, strategies for engaging in such discussions, realizing that one’s views matter as do all in a democracy); (ii) *cross-curricular* (e.g., language and communication skills, confidence and interpersonal skills, higher-order dialogic and thinking skills, information-processing, reasoning, enquiry, creative thinking, and evaluation skills); and (iii) *civic behavior* (greater political interest, pro-democratic values, increased political engagement, more civic knowledge, greater interest in discussing public affairs out of school, more likely to say, they will vote and volunteer as adults) (Huddleston & Kerr, 2015, p. 14–15, our emphasis).

Due to the documentary nature of this research (which does not involve observations of people interacting with the analysis materials), the justifications cited by Huddleston and Kerr (2015) were adapted by two analysts (first and second authors of this manuscript) towards identifying key elements that could properly answer our research questions.

Initially, the analysts separately selected the appropriate justification (if any) (a-i, a-ii, b-i, b-ii, b-iii) for the study and individually identified key elements within the previously selected justifications, thus creating categories of analysis. Finally, they compared and agreed on the selected key elements through discussions.

The categorization resulted in an investigation of the following six key elements: political, social, economic, moral, civic and productive exposure of different opinions, ideas, discourses and/or knowledge, and the elements that oppose the fear discourse – which refer to (a-i), (a-ii) and (b-i) justifications.

The literature points to the political dimension of controversy, since it crosses the interests and positions of institutions, including those of a governmental nature – which is especially evident in the Covid-19 pandemic and requires global efforts in crisis management (McIntyre, 2018; World Health Organization, 2020). Therefore, the political elements analyzed cite relevant entities in the local and/or global political scenario, legislative measures, and decision-makers.

Social elements in the exhibits concerned the impact of the pandemic on life in society or the civic duty of its citizens during the event, reinforcing the potential of museums for the exercise of citizenship. On the other hand, economic elements, also associated with the socio-political action that permeates the controversial science topics – from the perspective of the resources necessary for the production of knowledge and the impacts generated (Pedretti & Iannini, 2020) – refer to infrastructure, processes, technologies, and funding.

The socio-scientific controversy can also enable moral reasoning on science topics (Pedretti & Iannini, 2020). According to Zabala (1995), pp. 45, our translation), moral elements favor the learning of attitudinal contents, divided into values – “principles or ethical ideas that allow people to judge behavior and its meaning”, attitudes – “relatively stable tendencies or predispositions of people to act in a certain way”, and norms – “standards and rules of behavior to be followed in certain situations by all members of a social group” – considered in the perspective of such definitions for this analysis.

The civic and productive exposure of different opinions, ideas, discourses, and/or knowledge regards moments in which the exhibits (i) promote a clash of ideas through provocations for the visitor to take a stand, (ii) disseminate the positions taken by themselves, (iii) and give voice to “minority” groups towards breaking with prevailing stereotypes and paradigms and compensating for unilateral communications made by the media (Huddleston & Kerr, 2015). The latter aspect is especially relevant in view of a new generation of museums with the potential to promote debates and more progressive visions of science literacy – i.e., knowledge “of” and “about” science – and reflections on the importance of scientific evidence, argumentation, tolerance, and respect for individuals with different cultures and views (Pedretti & Iannini, 2020).

Elements that oppose the fear discourse concern moments in which controversy is assured in the exhibits as something common to everyday social life, whose discussion underpins democratic education and, therefore, should not be feared (Hess, 2009; Huddleston & Kerr, 2015). This approach becomes relevant in the face of the discourse of fear, in which certain institutional communications sensitize their audiences through visual and/or textual mechanisms that illustrate the catastrophic consequences of anti-scientific positions, strategies also used by denialist communities for exerting opposite effects (Shimizu, 2020).

### Text, audio, and visual contents

The survey on elements common to the teaching of controversial issues considered each slide a unit of analysis (UA), from the cover to the credits, in which text, audio, video, and/or image contents were investigated – the two latter were grouped together as visual contents. The exhibits, in their entirety, were taken as units of context (UC).

The basis for the categorization was the observation of the exhibits’ multimodality, i.e., the integration of different semiotic modes (e.g., combining written discourse with layouts, typographies, and illustrations), generating hybrid forms of communication (van Leeuwen, 2015). Although technology has enabled the customization of content and, therefore, expanded the idea of multimodality, those three categories of analysis were chosen because of their clear distinguishability, facilitating, for example, the analysis of videos with still images.

The categorization follows the concept of communicative act. In this sense, the information represented by audio, video, or text in the exhibits could still convey a complete message among the interlocutors through means and signs (Bordenave, 2017). All forms of written representations were considered texts; every voice speech was assumed an audio content and any imagetic representation, either dynamic (e.g, video recordings), or non-dynamic (such as still images), was classified as visual.

### Inter-rater agreement

The analysis of each exhibit was conducted individually by two analysts towards identifying the recurrence of elements related to the teaching of controversial science topics (political, social, economic, and moral ones, elements that oppose the fear discourse, and civic and productive exposure of different opinions, ideas, discourses, and/or knowledge) in the contents of the exhibits (visual, textual, and audio), detailed below. The agreement between the analysts was calculated by Cohen’s kappa coefficient (K). Cohen’s kappa is a statistical parameter commonly used to verify inter-rater agreements. It is more consistent than a simple percentage calculation, since it takes into account the agreement reached by chance (Cohen, 1960). Kappa varies between 0 and 1 and can be interpreted according to Landis and Koch (1977) as follows: poor agreement (K < 0); slight agreement (0 ≤ K ≤ 0.20); fair agreement (0.21 ≤ K ≤ 0.40); moderate agreement (0.41 ≤ K ≤ 0.60); substantial agreement (0.61 ≤ K ≤ 0.80); almost perfect agreement (0.81 ≤ K ≤ 1.00). Interestingly, the values calculated for Cohen’s kappa in this analysis were greater than 0.80 for all inter-rater agreements and non-agreements in scores were resolved through discussions.

Below are the results from the analysis of those elements in the contents of Coronacene – Thoughts in Times of Pandemic, COVID-19 – Mass manufacturing a vaccine, and Field in focus: predicting pandemics exhibits.

## Results and discussion

Although Google Arts & Culture platform maps almost all science museums and is a reference for museums of all areas of knowledge, especially in view of the growth of online exhibits, the contribution of STEM institutions to promoting virtual visits is still barely observable on it. Such a fact has opened a range of reflections on the underfunding of science museums for the maintenance of online communications (Kemp, 2021), platforms’ biases behind the digitalization of art and culture (Kizhner et al., 2021), absence of socio-scientific controversy in the collections of those institutions (Colombo Junior & Marandino, 2020), and a view of science that minimizes its sociocultural dimension (Davies et al., 2019).

The three online exhibits prepared by STEM institutions, which are the focus of this study, are detailed in what follows.

### Online exhibits

#### Coronacene – thoughts in times of pandemic (1)

Conceived by the Development and Management Institute in partnership with Estúdios Globo, GloboNews, and Fiocruz, Coronacene is a temporary exhibit of the Museum of Tomorrow *–* an institution founded in Rio de Janeiro, Brazil, in 2015, with the help of public and private authorities. The institution aims to provide “ideas, explorations, and questions about the time of great changes in which we live and the different paths opened up for the future”. The exhibit, made possible by the Culture Incentive Law and consisting of 37 slides, was launched in March 2021 and curated by Luiz Alberto Oliveira, Leonardo Menezes, and Eduardo Carvalho and showed the impacts of the pandemic on the role of science, struggle, and mourning.

In this perspective, it mixes texts (in English, Portuguese, and Spanish), statistical data, images, videos (2D and street views), and audio and shows both the physical space of the institution and tourist and everyday places around the world. It also displays testimonies (from a virologist and an indigenous leader), virus models, laboratories and scientific instruments (e.g., microscopes, computers, and flasks), and people (anonymous ones, visitors, indigenous ones, families, students, victims, teachers, cleaning professionals, health professionals, drivers, food delivery professionals, and scientists).

#### COVID-19 – mass manufacturing a vaccine (2)

The exhibit is part of the collection of the Museum of Engineering Innovation, a virtual institution created by the UK’s Royal Academy of Engineering and maintained by technical professionals, engineers, designers, and computer scientists in partnership with societies, companies, and universities. The collections, available in English on Google Arts platform since November 2020 under the label “this is engineering”, showcase engineering aspects not obvious in everyday life and inspire the next generation of professionals.

Twenty-seven slides show the work of a team of engineers at King’s College London responsible for the development of a large-scale production process for RNA vaccines, called the factory-in-a-box, against Covid-19. Texts and images compose the narrative, illustrating and describing scientific instruments (Eppendorf flasks, Petri dishes, among others), products (factory-in-a-box device and vaccines), virus models, laboratories, and people (team of engineers formed by professors and graduate students).

#### Field in focus: predicting pandemics (3)

The third exhibit, containing 40 slides, was organized by the Smithsonian Institution – a group founded in 1846 by the US Congress to value culture and science – in partnership with Myanmar ministries. With offices in Washington, United States, the Smithsonian organization has an international complex of museums, research centers, and cultural, educational, and zoological parks maintained with government and private fundings. The exhibit, available in English, retrieves a series of videos from the institution and adds elements from January 2020 for illustrating the work of scientists and government and society members in protecting animal species and predicting pandemics, including those caused by coronavirus.

Set in Myanmar, the online exhibit features texts, images (photographs of viruses obtained by scientific equipment, researchers, people, and animals), and videos (2D and 360°). The pandemic theme is explored in its emergence, highlighting the relationship between wild animals and diseases, including Severe Acute Respiratory Syndrome (SARS). Although old materials had been gathered from the institution and mixed with current images of coronaviruses, the exhibit took shape on the platform in the face of the current pandemic, especially to illustrate the birth of pandemics in nature – and not in the laboratory, as certain controversial speeches about Covid-19 dedicated themselves to affirming.

### Frequency of the contents and elements identified

**Fig. 2:**
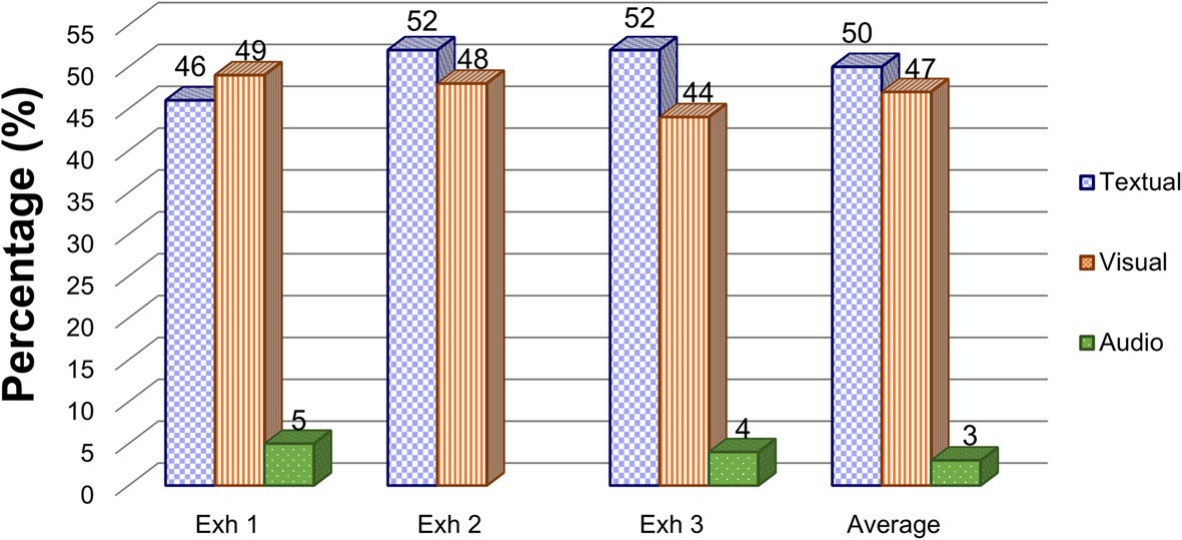
Quantification of textual, visual, and audio content by the Units of Analysis (UA)

Figure 2 displays the recurrence of textual, visual, and audio contents, as well as elements related to the teaching of controversial science topics in the exhibits. Each slide can concomitantly show more than one type of content, so that the number of UA of the three exhibits overlaps the total number of slides. The inter-rater reliabilities of the recurrence of the exhibits contents, calculated by Cohen’s kappa, were 0.86 (Exh 1), 0.92 (Exh 2), and 0.83 (Exh 3) – all reached the maximum level (Landis & Koch, 1977).

According to Fig. 2, textual content was the most recurrent resource, followed by visual and audio ones (50%, 47%, and 7% on average, respectively). In exhibits 2 and 3, the textual content was similar to popular science texts, since journalistic lead, explanatory procedures, boxes, eye-catching titles, among other aspects, were used for the dissemination of information (Vieira, 2007). According to Majetic & Pellegrino, 2018, and Tuten & Temesvari, 2013, popular science texts have mediated innovative didactic strategies that aim to provide skills from public communication of science and technology, such as critical thinking, information literacy, and oral and written expressions. The use of other textual types rather than the formal teaching of STEM is observed especially in exhibit 1, which included poems, revealing humanistic aspects of the scientific theme.

On the other hand, less frequent audio and video contents enabled exhibits 1 and 3 to be more interactive, making the Internet user be part of the experience, especially through the use of different online media formats (Bardoel & Deuze, 2001; Schultz, 2000). As an example, the museums incorporated street views and 360° video tools into their slides so that the visitor could explore the exhibit site even in front of a computer screen, thus opening up a range of interpretations for the concept of “visitation”. Exhibit 1 gave voice to its interviewees through the inclusion of podcasts, providing a more intimate experience to the visitor (Lindgren, 2016).

Indeed, the choice of contents of those exhibits revealed the way issues that limit science museums with a face-to-face structure also apply to an online environment, especially regarding debates on the contemplative and interactive nature of the exhibitions, which call into question the possibility of the visitor acting as a mere observer or pusher of buttons (Cazelli et al., 2003; Pedretti & Iannini, 2020). In digital format, information competes for the Internet user’s attention (Zulli, 2018) and, therefore, betting on attractive and interactive resources can facilitate the retention of the visitor in that environment, since it is enough to close the browser tab for leaving the exhibit.

**Fig. 3:**
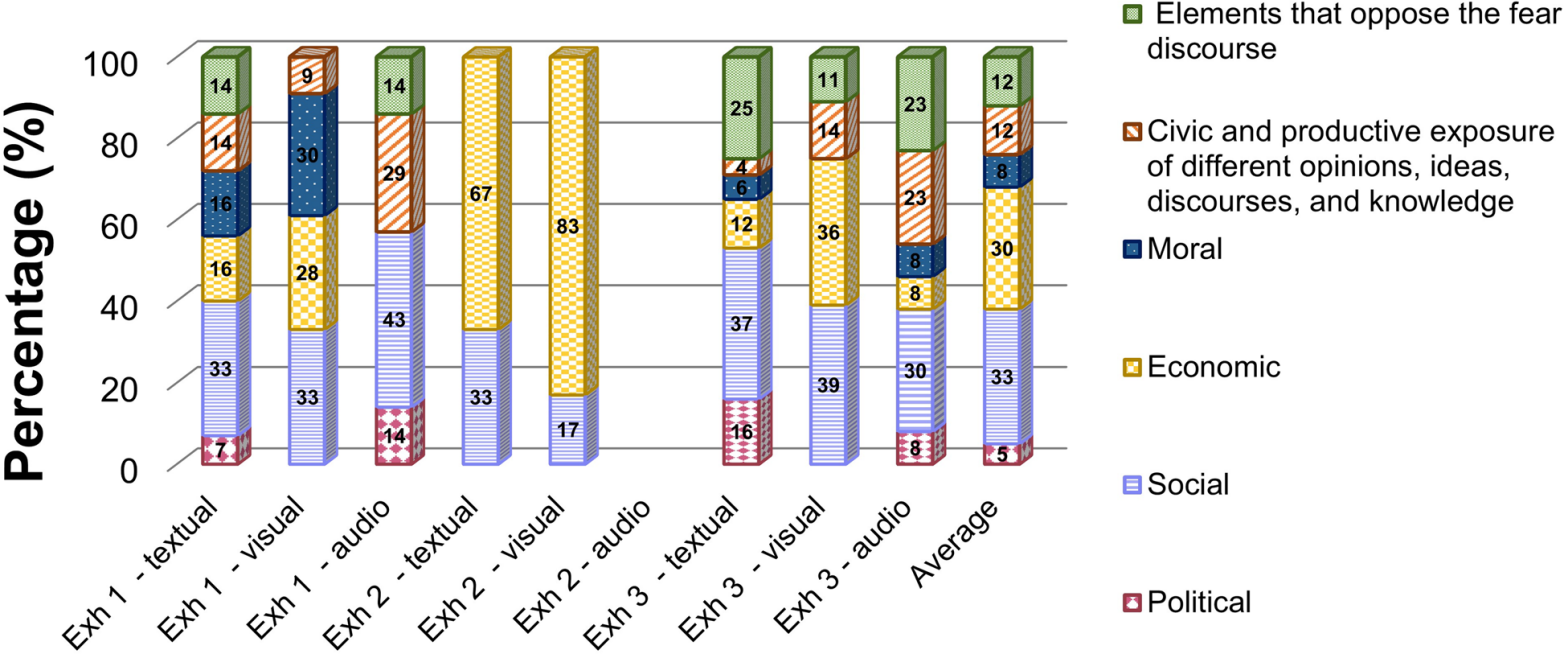
Frequency of elements about teaching of controversial issues in the exhibits per materials

Figure 3 shows data on the frequency of the elements commonly present in the teaching of controversial issues per online exhibit on Covid-19 prepared by STEM institutions, evidencing their recurrence in the different types of content used in the narratives (textual, visual, and/or audio). The inter-rater reliability of the analysis, calculated by Cohen’s kappa, was substantial for exhibits 1 and 3 (K = 0.74 and K = 0.69, respectively) and almost perfect for exhibit 2 (K = 0.81), according to Landis and Koch (1977). Such elements are not exclusive, i.e., a same UA can contain references to more than one element, and they are also intrinsically connected with the approach chosen by the exhibits.

Social elements were the most recurrent in all contents of exhibit 1 (33% present in textual contents and 33% and 43% in visual and audio ones, respectively) due to the focus given to the socio-scientific controversy of COVID-19 pandemic, which covered mainly the impacts of coronavirus on the society. Figure 3 also shows how the disease affected the daily lives of people and different social groups, the economy, and the routine of cities. Therefore, the significant and well-distributed presence of political, economic, and moral elements, elements that oppose the fear discourse, and the civic and productive exposition of different opinions, ideas, discourses and/or knowledge can be observed in its contents.

In contrast, exhibit 2 focused only on economic and social elements. The first stands out and corresponds to 83% and 67% of visual and textual contents, respectively, due to the perspective adopted by the museum, which portrays a methodology for a mass production of vaccines against the coronavirus. As a result, the social element was also considered to showcase the importance of engineers in dealing with the pandemic (33% of textual content and 17% of visual one).

Similarly to exhibit 1, exhibit 3 showed a predominance of social elements in the three types of content, followed by a civic and productive exposition of different opinions, ideas, discourses, and/or knowledge, and the elements that oppose the fear discourse – the latter two present mainly in text and audio contents. Such recurrence is associated with the fact the museum focuses on the importance of science and collaboration among governments, citizens, and scientists to map and identify new viruses, thus preventing the occurrence of future pandemics. In addition to the social aspect, the theme enabled the emergence of political, economic, and moral discussions.

In general, social and economic elements were the most frequent in the three exhibits (33% and 30% on average, respectively), in contrast to political ones (5% of the contents identified, on average). The results regard the approaches chosen by the museums to address the controversial topics, discussed in what follows.

#### Political, social, economic, and moral elements

Regarding the political elements, present only in exhibits 1 and 3, the museums aimed to show mainly the relevance of cooperation among the various organizations for solving the Covid-19 pandemic (e.g., WHO), mentioning, among other aspects, health guidelines, government measures to support research, and the impacts of political events on the pandemic scenario:

Global Health Program researchers work closely with resident scientists — and collaborate with Myanmar’s Ministry of Agriculture, Livestock and Irrigation; Ministry of Health and Sports; and Ministry of Natural Resources and Environmental Conservation — to collect and analyze biological samples from wildlife and the humans they come in contact with (Exhibit 3, slide 5).

The community that surrounds the non-formal and informal teaching spaces often does not perceive the political dimension attached to them (Allen & Crowley, 2014; Lewenstein, 2016); consequently, they do not exercise their political role in society, which resides in their being attentive to.

(...) a multiple range of actors and processes that constantly re-examine the social aspect from its interactions, not to configure a heroic narrative or to situate it as a unique agent of change, but rather to unveil its complexities, alter its meanings, and relate it to other social agents. In this light, the position of the museum in relation to its interaction with networks of social agents, citizenship, and the various institutions, practices, and actions must be analyzed (Montero, 2012, p. 79, our translation).

Such a lack of perception of the political dimension is evidenced in exhibit 2, which focused on the economic elements associated with the theme from the following statement: “To bring Covid-19 under control with a vaccine, we will need to vaccinate about 60% of the almost 8 billion of people on Earth” (slide 6). Economy is projected in exhibit 2 due to its focus on a method of a large-scale manufacture of vaccines for Covid-19. However, as pointed out by Shimizu (2020), the issue of vaccination concerns not only infrastructure, but also social, cultural, political, and historical aspects. In this sense, the museum environment and the controversial debate lose their potential to promote democratic education and a sense of citizenship (Pedretti & Iannini, 2020) to be considered by curators, teachers, and instructors in view of the activities offered.

In the other exhibits, the economic elements were mostly associated with the employment issue (exhibit 1) and the scientific research infrastructure (exhibit 3). In those cases and in line with the approach chosen by the institutions, the intersection of those aspects with the socio-political dimension of the theme was more explicit in contents such as.

Transformed societies. Have you ever stopped to think you might go through a pandemic? For many, the answer is no. In 2020, companies went bankrupt, business closed its doors and our routines were interrupted due to the new coronavirus. Though it seems like a science fiction film, this really happened. Brazil, 14.1 million unemployed. 3rd quarter/2020, IBGE, Instituto Brasileiro de Geografia e Estatística/Brazilian Institute of Geography and Statistics (Exhibit 1, slide 15).

Social elements were present in all exhibits, associating human behavior with the development of the pandemic in texts and images. Exhibit 1 portrayed the consequences of the disease for the society, showing its impacts on people’s daily lives, in the forms of communication and interaction with the world:

COVID-19 disproportionately affects groups who are neglected by society. It is essential to face these social problems for tackling this challenge. Otherwise, we will never be able to fully overcome the 2020 pandemic or other future ills, even with vaccines or medications (Exhibit 1, slide 28).

Exhibit 2 highlighted the researcher’s civic duty of seeking solutions to health issues, and in exhibit 3, the social elements focused on the impacts of science on studies of the nature and prediction of pandemics. By approaching those elements, museums favor reflections on the social dimension of science, sometimes suppressed throughout STEM careers. They are also aligned with the science, technology, and society (STS) movement, which aims at teaching science that forms citizens capable of making critical decisions and envisioning the implications of science and technology in everyday social life (Mansour, 2009).

**Fig. 4:**
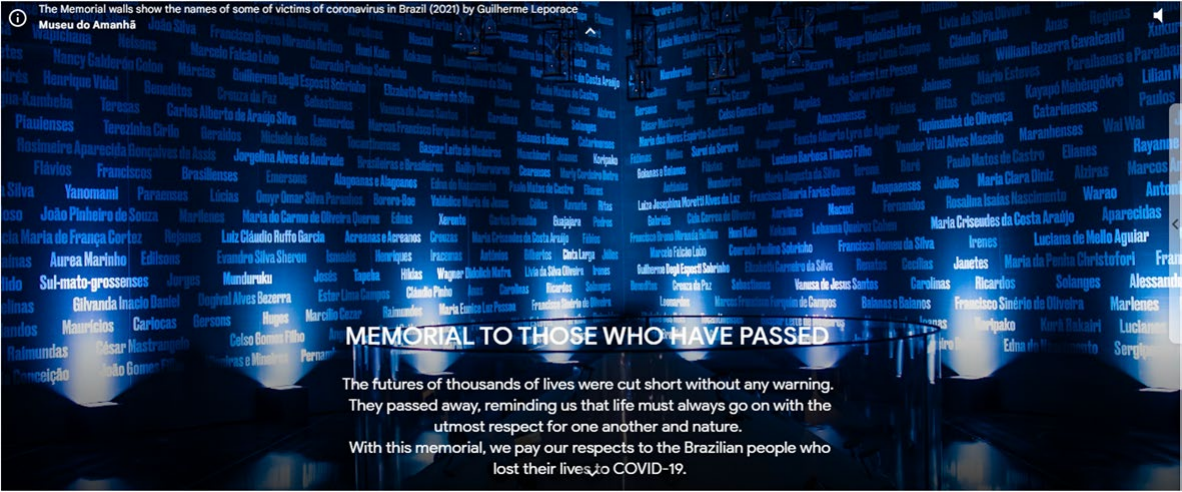
Excerpt from exhibit 3 that refers to moral elements

Finally, the moral element emerged in some moments of exhibits 1 and 3. In the former, it stood out in the memorial dedicated to the victims of Covid-19 in Brazil (Fig. 4), whereas in the third, the attitudes and values of organizations towards respecting the human-wild animal relationship and concomitantly fighting the pandemic were discussed:

At times, it may also be difficult to ask local participants to wear the protective equipment when they have long interacted with these animals without it. Ultimately, the team’s goal is to put as little stress as possible on their partners in the field, the local community members they interact with and the animals they sample (Exhibit 3, slide 29).

By highlighting the moral character of socio-scientific controversies, museums show aspects external to science, i.e., those that dialogue with personal values, behaviors, and attitudes (Pedretti & Iannini, 2020), which, according to Meyer (2009), is a potential for museums to not only address a controversial topic, but also generate controversy.

In general, bringing debates over social, political, economic, and moral issues to the exhibits contributed to the discussion of social events in the light of evidence and to teaching, favoring a more “real” and “human” science in the eyes of the public (Pedretti & Iannini, 2020). Such debates show characteristics of a science that deals with problems faced in everyday life and that moves away from an absolute truth. From this perspective, exhibit 2 highlighted the provisional nature of science, i.e., that science is mutable, favoring the emergence of new knowledge as new evidence rises (Sotério, 2022), especially in the face of an unprecedented disease:

(...) Currently, we don’t even know how long immunity from a vaccine will last because Covid-19 is so brand new. We may need one or two doses, or it could be needed every year like the flu jab (Exhibit 2, slide 6).

By approaching those aspects, museums are no longer spaces for the presentation of a finished and immutable scientific knowledge as a transforming agent of reality concomitantly affected by its demands. They increasingly assume the role of contemporary agoras (Pedretti & Iannini, 2020), acting as a “safe space for difficult discussions” (Science Center World Congress, 2008, p. 1).

#### Elements aimed at civic and productive exposure of different opinions, ideas, discourses, and/or knowledge

The civic and productive exposition of different opinions, ideas, discourses, and/or knowledge in the exhibits prepared by STEM institutions is evident in the proposition of provocative questions, giving space to the confrontation of ideas without distancing itself from the scientific discourse. Such elements appeared only in exhibits 1 and 3; they can be included in museum exhibits precisely through provocative questions, favoring public engagement in discussions on topics considered complex and leading to a confrontation of the visitor’s personal values with scientific evidence (Pedretti & Iannini, 2020). Exhibit 1, in which such elements are more recurrent, showed both texts and audiovisual contents that promoted those aspects.

Turning point. Microorganisms have always shaped human history. Just like the Black Death, the Spanish flu, and HIV. In 2020, the new coronavirus affected us all. Will life ever be the same as it was before? Do we want that? Will this struggle prepare us for other global challenges, such as the climate change? (Exhibit 1, slide 3).

In fact, our questioning on whether we want the same life as the one we had before leads to a contraposition of ideas, thus opening paths for reflections on the “old” and “new” normal. The dilemma refers to what Kuhn proposed when weaving relations between the already established (normal) science that responds satisfactorily to the current paradigm but which, in the face of events whose solutions are no longer satisfactory, gives rise to a new (revolutionary) science that, with time, tends to become a normal one again (Kuhn, 1970). Such a behavior reflects the way moments of rupture provide scientific advance. Indeed, we tend to leave the pandemic towards a daily life with more advanced technology in which, for example, vaccines are produced in record times (Lurie et al., 2020). Concomitantly, we will also get used to a way of existence with at least 6 million fewer people in the world — WHO data from April, 2022.

On the other hand, the civic clash of ideas appears through the taking of a position by the STEM institutions, within a controversial topic. As an example, exhibit 1 cited the origin of the Sars-Cov-2 virus without giving space to the negationist discourse that limits the subject, but positioning itself alongside the scientific evidence:

From virus to pandemic. The first records of the SARS CoV-2 virus, which causes COVID-19, are from the city of Wuhan, China in 2019. The initial outbreak among regulars of an exotic-animal wet market suggests that the disease was transmitted by an animal (Exhibit 1, slide 12).

**Fig. 5:**
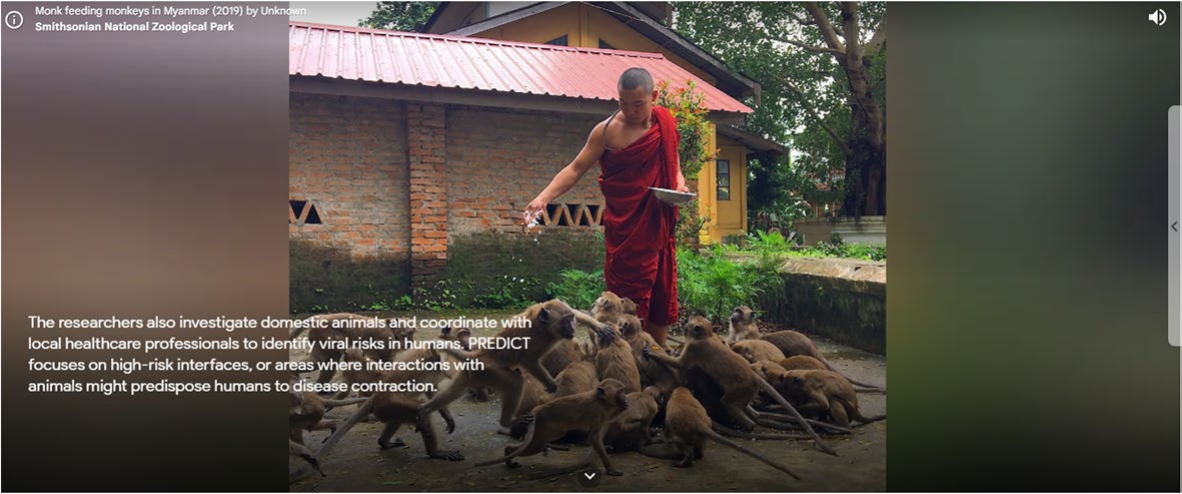
Civic and productive exposition of different opinions, ideas, discourses, and/or knowledge (exhibit 3)

Such a position has become quite emblematic regarding the Covid-19 theme, thus leading to a clash between scientists – armed with evidence of the emergence of the disease from animal vectors – and conspiracy theories about the creation of the virus in the laboratory. The same issue was portrayed in exhibit 3 towards destigmatizing the human-wild animal relationship in the light of scientific knowledge (Fig. 5), compensating for unilateral communications made by the media that fomented xenophobic discourses and unregulated slaughter of those species as a disease containment measure (Abutaleb & Harris, 2021; Budhwani & Sun, 2020; Maron, 2021; Sacramento et al., 2020).

Exhibit 3 also brought perceptions of different experts (e.g., ecologists, veterinarians, and social scientists) on the pandemic at different moments of the narrative, offering the visitor a range of knowledge equally relevant to solving the problem at hand. The inclusion of such elements has contributed positively to the dissemination of undistorted views of science through non-formal teaching spaces, since they show collaboration among groups, teamwork, and multidisciplinarity (Pérez et al., 2001).

According to Pedretti and Iannini (2020), the exposure of controversial topics can potentially develop argumentative and critical skills fostered by exhibits in situations such as those aforementioned. However, museums occasionally miss such an opportunity by suggesting unique answers and/or interpretations to questions raised. As an example, although exhibit 1 has opened space for reflections on the future desired in a post-Covid-19 pandemic scenario, it has delivered an answer that speaks for the collective: “When all this is over, we want the Tomorrow that is more sustainable, less unequal” (Exhibit 1, slide 7).

#### Elements that oppose the fear discourse

Elements on this topic were detected only in exhibits 1 and 3, and both elements that oppose the fear discourse, aligned with the desirable elements in teaching controversy (Huddleston & Kerr, 2015), were identified.

Exhibit 1 used elements that oppose the fear discourse to highlight the way pandemics are naturally recurrent in the history of life in society and, therefore, should be seen as a learning period for future pandemics, which is facilitated by scientific development, as shown in:

Science is the protagonist. During the pandemic, science took its place at the forefront of the response to the coronavirus, developed health guidelines that slowed the infection rate, and prevented healthcare systems from becoming quickly overwhelmed. Now, scientists work to develop diagnostics and healthcare products (Exhibit 1, slide 25).

**Fig. 6:**
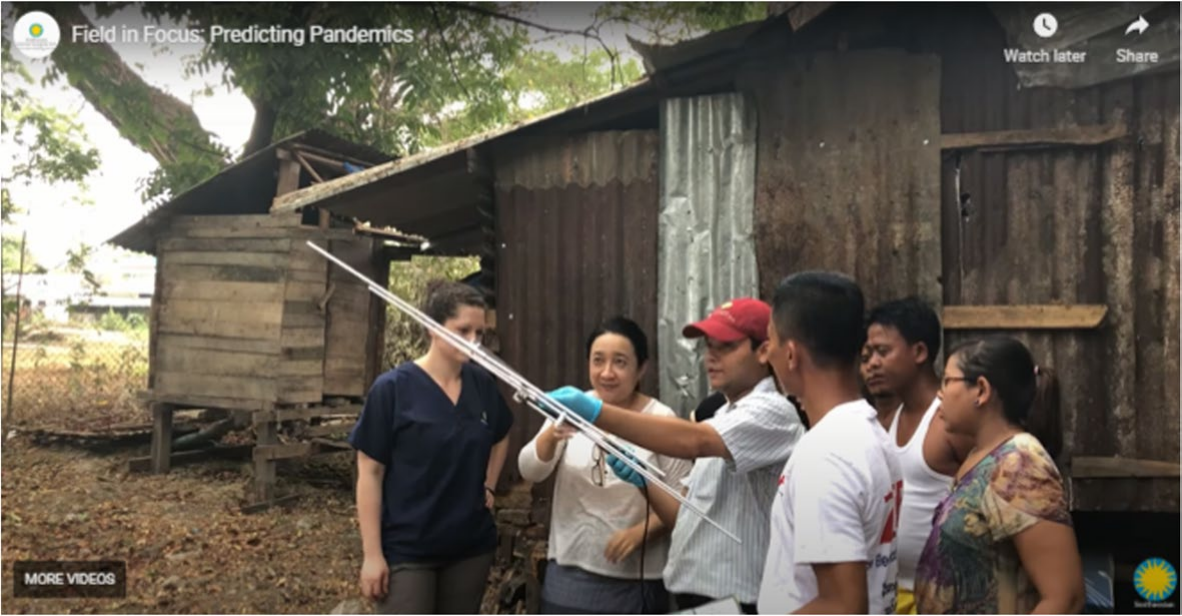
Excerpts from exhibit 3 that refer to elements that oppose the fear discourse

Similarly, exhibit 3 reinforced the need to discuss the prevention of pandemics through science, which is possible only with the collaboration of local communities and government. In this case, elements that oppose the fear discourse point to the destigmatization of the public debate on such a controversial topic by illustrating a community in which those groups operate together despite their differences (Fig. 6).

**Fig. 7:**
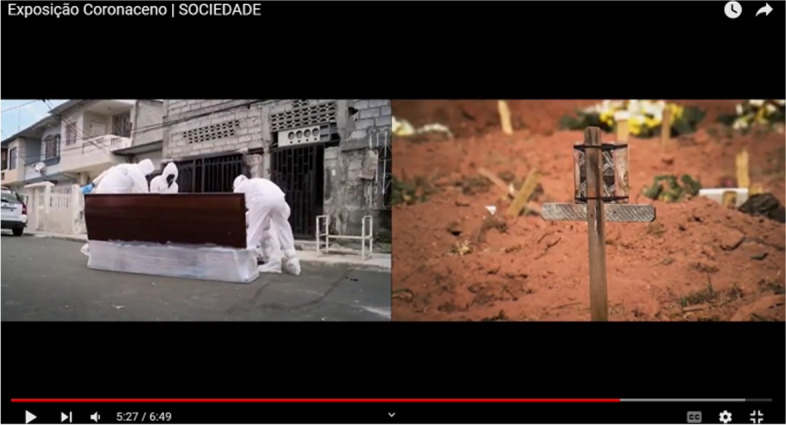
Excerpt from exhibit 1 that refers to the fear discourse

On the other hand, the fatality of the disease, materialized in a video showing coffins and graves (Fig. 7, referring to exhibit 1), promotes the audience’s awareness of the seriousness of the matter and the importance of searching for accurate information.

Evidently, Covid-19 has brought daily life closer to an imminent tragedy; however, the denialist discourse has also appropriated the theme of fear and catastrophe towards discrediting the facts. As an example, the president of Brazil, Jair Bolsonaro, started to defend the number of people recovered from the disease in order to omit the state of public calamity, alleging mental exhaustion of those who followed the “pessimistic data” (Lemos, 2020).

As highlighted by Shimizu (2020), fear articulates different positions in a controversial debate, both trust and distrust. In the latter case, exhibits may lose the potential to promote educational engagement, i.e., to attract visitors to an experience that results in learning (Pedretti & Iannini, 2020), thus moving away from the aforementioned elements, which they value for a teaching that destigmatizes debates on controversial issues.

## Conclusions

In general, the educational dimension was present in the online exhibits on Covid-19, covering the discussion on controversial issues of science topics at different levels regarding political, social, economic, and moral elements, elements that oppose the fear discourse, and civic and productive exposure of different opinions, ideas, discourses, and/or knowledge (Huddleston & Kerr, 2015).

The structure of those virtual educational spaces showed a predominance of textual content, a resource commonly used in traditional science teaching, and fewer uses of audio content. The multimediality allowed by Google Arts & Culture puts museums at an advantage over other competing media that do not have so many resources. However, an exacerbated use of a single type of content can cause online exhibits to lose their potential for multimedia instruction – i.e., the combination of pictures (still images, videos, or any other format) and words (printed or spoken texts) that leads to their cognitive representations to promote learning (Mayer, 2017).

Such a dynamic also reflects a discussion familiar to the history of science museums, in which the role of the visitor in an expository versus interactive exhibit is questioned. Exhibits 1 and 3, which extensively used different online media formats such as 360° videos, street views, and podcasts, promoted greater interaction and extrapolation of the “visitation” concept, thus making the visitor the center of their learning process in a self-guided adventure from one click. On the other hand, when only images and texts are used (as in exhibit 2), such possibilities are reduced, bringing the visitor closer to being a spectator. Digital interactivity, which competes with the retention of the Internet user’s attention (Bardoel & Deuze, 2001), must not be distanced from its educational purpose, so that it does not turn the visitor into a mere “pusher of buttons” (Cazelli et al., 2003; Pedretti & Iannini, 2020).

The political, economic, social, and moral elements revealed a correspondence between the theme and the social dimension in all exhibits (33% of the contents, on average), as expected from a controversial discussion that deals with themes that affect society in different spheres. On the other hand, the political issue was the least frequent in the narratives (5% on average) and superficially covered at certain times. Such a result is in line with studies that deal with the non-perception of the political dimension of non-formal and informal teaching spaces (Allen & Crowley, 2014; Lewenstein, 2016). In this sense, curators, teachers, and instructors must pay attention to the museum’s potential to promote democratic teaching and citizenship training based on controversial themes.

Undistorted conceptions of science and more progressive views of science literacy, desirable in a new generation of museums (Pedretti & Iannini, 2020), should be encouraged by curators and instructors. Such aspects were facilitated by the presentation of characteristics of science and the scientific process, such as provisionality, collaboration among groups, infrastructure requirements, and multidisciplinarity. The results were achieved from the insertion of social, moral, economic, and political elements and from the civic and productive exposition of different opinions, ideas, discourses, and/or knowledge in the exhibits.

Compensation for unilateral media communications, which is beneficial to public debates (Huddleston & Kerr, 2015), occurs when cultural and customs differences, beliefs, and opinions on a controversial subject are guaranteed in museums, as observed in the civic and productive exposure of different opinions, ideas, discourses, and/or knowledge. Since the exhibits were curated by STEM institutions, the elements led to reflections and the taking of a position on the Covid-19 topic without departing from a scientific discourse. Such a result corroborates the potential for scientists and STEM institutions active in social networks to mitigate the effects of misinformation caused by the Covid-19 infodemic (World Health Organization, 2020).

Regarding elements that oppose the fear discourse, exhibit 3 revealed an effort to naturalize the controversial discussion and illustrate the collaboration of different social groups towards thinking of joint solutions. On the other hand, exhibit 1 gave way to the fear discourse through mechanisms of awareness on the severity of the disease and the role of science in the face of fatality. Fear articulates both pro and anti-science positions (Shimizu, 2020), which may reduce the potential of educational engagement by museums, due to movements observed throughout the pandemic that tried to associate debates on controversial themes with mental exhaustion. Fatality should not be ignored; however, mechanisms must be created so that museums can provide an inviting environment for the resolution of challenging issues, given the diversity of thoughts and behaviors that coexist in a society (Huddleston & Kerr, 2015).

It is noteworthy that the results reported here on the types of content (visual, textual, or audio) analyzed in the three exhibitions created by STEM institutions are related to the topic of Covid-19. Therefore, different findings are likely to be observed in analyses of exhibits that address other socio-scientific issues, such as climate change or evolution.

This study on controversial issues of science topics showed “that our view of scientific content knowledge is dependent on our culture, for example our norms, values and worldviews, and it is dependent on the time we are living in” (Sjöström & Eilks, 2018, p. 67). However, the presence of exhibits organized by STEM institutions is still not very recurrent on Google Arts & Culture platform, when compared to other areas of knowledge. In this sense, a way has been opened for reflections on the sociocultural character of science and technology: what is preventing us from connecting hard sciences to “Arts & Culture”?

Other limitations regarding the low publication rate of Covid-19 exhibitions by STEM institutions on the analyzed platform may be related to the posting of subjects to Google approval, the need for the exhibition belonging to an organization with its own institutional website and email address, and the exhibit preset formats of slideshows (Google Arts & Culture Platform Help, 2022). Specific analyses on the platform’s advantages and disadvantages incorporate the current literature in the field (Proctor, 2011; Cowin, 2020; Kizhner et al., 2020; Verde & Valero, 2021).

In contrast, the adhesion of museums to digital environments was intensified during the pandemic, for many, as a matter of survival (Vadja, 2020), increasing the number of online visitors in 2020, but also required the existence of a “digital staff” and financial investments from museums. The challenges for the maintenance of such institutions have become a public concern that urges government investments, since museums house collections related to the past, present, and future of humankind and many organizations lack the necessary infrastructure to publish online exhibits (International Council of Museums, 2020; Network of European Museum Organisations, 2020).

Future work should investigate facts not addressed in this documentary study and that refer to the analysis of visitors’ interactivity with the museums’ online exhibitions towards identifying other elements pointed out by Huddleston and Kerr (2015) regarding cross-curricular aspects and civic behavior. Another limitation to be addressed is the teaching of controversial science topics based on platforms that provide quantitative data about number of clicks, views, and visitor interaction time in each exhibition, currently not available on Google Arts & Culture for its visitors. Comparisons on the approach to the Covid-19 pandemic by STEM museums in online exhibits and other competing media – such as independent websites and social networks – can also be made for guiding future actions for online exhibitions created by STEM institutions and fostering discussions on socio-scientific topics.

## Data Availability

All data analyzed in this study are available from the corresponding author on reasonable request.

## Abbreviations

HIV: Human Immunodeficiency Virus
IBGE: Brazilian Institute of Geography and Statistics
ICOM: International Council of Museums
RNA: Ribonucleic acid
SARS: Severe Acute Respiratory Syndrome
STEM: Science, Technology, Engineering, and Mathematics
UA: Unit of analysis
UC: Unit of context
WHO: World Health Organization

## References

[CR1] Abutaleb, B. Y., & Harris, S. (2021). *Trump administration’s hunt for pandemic ‘lab leak’ went down many paths and came up with no smoking gun*. https://www.washingtonpost.com/national-security/us-intelligence-wuhan-lab-coronavirus-origin/2021/06/15/2fc2425e-ca24-11eb-afd0-9726f7ec0ba6_story.html. Accessed 25 Mar 2022.

[CR2] Allen, L. B., & Crowley, K. J. (2014). Challenging beliefs, practices, and content: How museum educators change. *Science Education*, *98*(1), 84–105. 10.1002/sce.21093.

[CR3] Arnold, K. (1996). Presenting science as productor as a process: Museums and the making of science. In S. Pearce (Ed.), *Exploring science in museums*. London: The Athlone Press.

[CR4] Bardoel, J., & Deuze, M. (2001). “Network journalism”: Converging competences of old and new media professionals. *Australian Journalism Review*, *23*(2), 91–103.

[CR5] Blaney, J. R., & Hunt, S. K. (2021). COVID-19 internet responses from the state broadcast associations: Community, industry, and extent of spread. *Journal of Radio & Audio Media*, 1–14. 10.1080/19376529.2020.1870466.

[CR6] Blustein, D. L., Duffy, R., Ferreira, J. A., Cohen-Scali, V., Cinamon, R. G., & Allan, B. A. (2020). Unemployment in the time of COVID-19: A research agenda. *Journal of Vocational Behavior*, *119*(103436), 1–4. 10.1016/j.jvb.2020.103436. 10.1016/j.jvb.2020.103436 PMC7206417 32390656

[CR7] Bordenave, J. E. D. (2017). *O que é Comunicação*. São Paulo: Editora Brasiliense.

[CR8] Brotman, J. S., Mensah, F. M., & Lesko, N. (2011). Urban high school students’ learning about HIV/AIDS in different contexts. *Science Education*, *95*(1), 87–120. 10.1002/sce.20405.

[CR9] Budhwani, H., & Sun, R. (2020). Creating COVID-19 stigma by referencing the novel coronavirus as the “Chinese virus” on twitter: Quantitative analysis of social media data. *Journal of Medical Internet Research*, *22*(5), 1–7. 10.2196/19301. 10.2196/19301 PMC7205030 32343669

[CR10] Butler, S. (1992). *Science and technology museums*. Leicester: University Press.

[CR11] Casero-Ripollés, A. (2021). The impact of Covid-19 on journalism: A set of transformations in five domains. *Comunicação e sociedade*, *40*, 53–69 http://journals.openedition.org/cs/5890.

[CR12] Cazelli, S., Marandino, M., & Studart, D. C. (2003). Educação e comunicação em museus de ciência: aspectos históricos, pesquisa e prática. In G. Gouvêa, M. C. Leal, & M. Marandino (Eds.), *Educação e Museu: a construção social do caráter educativo dos museus de ciências*. Rio de Janeiro: Editora Access.

[CR13] Cohen, J. (1960). A coefficient of agreement for nominal scales. *Educational and Psychological Measurement*, *20*(1), 37–46. 10.1177/001316446002000104.

[CR14] Colombo Junior, P. D., & Marandino, M. (2020). Museus de ciências e controvérsias sociocientíficas: reflexões necessárias. *Journal of Science Communication América Latina*, *03*(1), A02. 10.22323/3.03010202.

[CR15] Cowin, J. B. (2020). Digital worlds and transformative learning: Google expeditions, Google arts and culture, and the merge cube. *International Research and Review*, *10*(1), 42–53.

[CR16] Davies, S. R., Halpern, M., Horst, M., Kirby, D. A., & Lewenstein, B. (2019). Science stories as culture: Experience, identity, narrative and emotion in public communication of science. *Journal of Science Communication*, *18*(5). 10.22323/2.18050201.

[CR17] Delicado, A. (2004). *Para que servem os museus científicos? Funções e finalidades dos espaços de musealização da ciência*, *VIII Congresso Luso-Afro-Brasileiro de Ciências Sociais* (pp. 1–17) https://www.ces.uc.pt/lab2004/pdfs/AnaDelicado.pdf. Accessed 25 Mar 2022.

[CR18] Delicado, A. (2007). *“What do scientists do” in museums: Representations of scientific practice in museum exhibitions and activities*, *The Pantaneto Forum* (p. 26) http://pantaneto.co.uk/what-do-scientists-do-in-museums-representations-of-scientific-practice-in-museum-exhibitions-and-activities-ana-delicado/. Accessed 15 Sept 2022.

[CR19] Domínguez, P. A. (2009). Espacios educativos y Museos de Pedagogía, Enseñanza y Educación. *Cuestiones Pedagógicas: Revista de Ciencias de La Educación*, *19*, 191–206.

[CR20] Elias, A., Ben, J., Mansouri, F., & Paradies, Y. (2021). Racism and nationalism during and beyond the COVID-19 pandemic. *Ethnic and Racial Studies*, *44*(5), 783–793. 10.1080/01419870.2020.1851382.

[CR21] Google Arts & Culture Platform Help. (2022). *About Google Arts & Culture.* https://support.google.com/culturalinstitute/partners/answer/4395223?hl=en&ref_topic=4387717. Accessed 13 Nov 2022.

[CR22] Hess, D. E. (2009). *Controversy in the classroom: The democratic power of discussion*. New York/Abingdon: Routledge (Taylor & Francis Group).

[CR23] Huddleston, T., & Kerr, D. (2015). *Teaching Controversial Issues Through Education for Democratic Citizenship and Human Rights (EDC/HRE)*. https://www.history.org.uk/secondary/resource/780/the-teach-report. Accessed 25 Mar 2022.

[CR24] International Council of Museums. (2020). *Museums, Museum professionals and COVID-19: Follow-up survey*. https://icom.museum/en/news/follow-up-report-museums-covid-19/. Accessed 25 Mar 2022.

[CR25] Kemp, S. (2021). *Digital 2021: a global overview report*. https://datareportal.com/reports/digital-2021-global-overview-report. Accessed 25 Mar 2022.

[CR26] Kizhner, I., Terras, M., Rumyantsev, M., Khokhlova, V., Demeshkova, E., Rudov, I., & Afanasieva, J. (2021). Digital cultural colonialism: Measuring bias in aggregated digitized content held in Google arts and culture. *Digital Scholarship in the Humanities*, *36*(3), 607–640.

[CR27] Kizhner, I., Terras, M., Rumyantsev, M., Khokhlova, V., Demeshkova, E., Rudov, I., & Afanasieva, J. (2020). Digital cultural colonialism: measuring bias in aggregated digitized content held in Google Arts and Culture. *Digital Scholarship in the Humanities*, 36(3), 607-640. 10.1093/llc/fqaa055.

[CR28] Kuhn, T. S. (1970). *The structure of scientific revolutions*. Illinois: University of Chicago Press.

[CR29] Landis, J. R., & Koch, G. G. (1977). The measurement of observer agreement for categorical data. *Biometrics*, *33*(1), 159–174. 843571

[CR30] Lemos, V. (2020). *“Placar da vida” do governo estimula negacionismo por omitir realidade trágica da covid-19, dizem cientistas*. São Paulo, BBC News https://www.bbc.com/portuguese/geral-52765075. Accessed 25 Mar 2022.

[CR31] Lerario, A. (2021). Languages and context issues of ICTs for a new role of museums in the COVID-19 era. *Heritage*, *4*(4), 3065–3080. 10.3390/heritage4040171.

[CR32] Lewenstein, B. (2016). *Public Engagement*. https://www.informalscience.org/news-views/public-engagement. Accessed 25 Mar 2022.

[CR33] Lindgren, M. (2016). Personal narrative journalism. *The Radio Journal - International Studies in Broadcast & Audio Media*, *14*(1), 23–41. 10.1386/rjao.14.1.23.

[CR34] Lurie, N., Saville, M., Hatchett, R., & Halton, J. (2020). Developing Covid-19 vaccines at pandemic speed. *New England Journal of Medicine*, *382*(21), 1969–1973. 10.1056/NEJMp2005630. 32227757 10.1056/NEJMp2005630

[CR35] Majetic, C., & Pellegrino, C. (2018). Building information literacy skills using science news media: Evidence for a hands-on approach. *Journal of College Science Teaching*, *48*(1), 83–91 https://www.jstor.org/stable/26491350.

[CR36] Mansour, N. (2009). Science-technology-society (STS). *Bulletin of Science, Technology & Society*, *29*(4), 287–297. 10.1177/0270467609336307.

[CR37] Marandino, M. (2008). *Educação em museus: a mediação em foco*. http://www.geenf.fe.usp.br/v2/wp-content/uploads/2012/10/MediacaoemFoco.pdf. Accessed 25 Mar 2022.

[CR38] Maron, D. F. (2021). *Surto de covid-19 em visons gerou lições preocupantes sobre a pandemia*. https://www.nationalgeographicbrasil.com/ciencia/2021/03/surto-de-covid-19-em-visons-gerou-licoes-preocupantes-sobre-a-pandemia. Accessed 25 Mar 2022.

[CR39] Mayer, R. E. (2017). Introduction to multimedia learning. In R. E. Mayer (Ed.), *The Cambridge handbook of multimedia learning*. Cambridge: Cambridge University Press.

[CR40] McIntyre, L. C. (2018). Post-Truth. Cambridge/London: The MIT Press.

[CR41] Meyer, M. (2009). *From ‘cold’ science to ‘hot’ research: the texture of controversy*. https://halshs.archives-ouvertes.fr/halshs-00417427/. Accessed 17 Sep 2022.

[CR42] Montero, J. R. (2012). Experiencias de mediación crítica y trabajo en red en museos: de las políticas de acceso a las políticas en red. *Revistas Museos*, *31*, 76–87.

[CR43] Museum of Engineering Innovation. (2020). *COVID-19 – Mass manufacturing a vaccine*. https://artsandculture.google.com/story/6wVBUjd3PCfXwQ. Accessed 13 Sep 2022.

[CR44] Museum of Tomorrow. (2021). *Coronacene – Thoughts in Times of Pandemic*. https://artsandculture.google.com/story/9gVhS6T56XCs0w. Accessed 13 Sep 2022.

[CR45] Nelson, J. L. (2020). The enduring popularity of legacy journalism: An analysis of online audience data. *Media and Communication*, *8*(2), 40–50. 10.17645/mac.v8i2.2736.

[CR46] Network of European Museum Organisations. (2020). *Survey on the impact of the COVID-19 situation on museums in Europe Final Report*. https://www.ne-mo.org/fileadmin/Dateien/public/NEMO_documents/NEMO_COVID19_Report_12.05.2020.pdf. Accessed 13 Sep 2022.

[CR47] Nielsen. (2020). *COVID-19: Tracking the Impact on Media Consumption*. https://www.nielsen.com/insights/2020/covid-19-tracking-the-impact-on-media-consumption/. Accessed 13 Sep 2022.

[CR48] Pastor Homs, M. I. (2009). Fields of action in non formal education. A suggested taxonomy. *Teoría de La Educación. Revista Interuniversitaria*, *11*, 183–215. 10.14201/2850.

[CR49] Pedretti, E., & Iannini, A. M. N. (2020). *Controversy in science museums: Re-imagining exhibition spaces and practice*. London/New York: Routledge (Taylor & Francis Group).

[CR50] Pedretti, E., Iannini, A. M. N., & Nazir, J. (2018). Exploring controversy in science museums: Non-visitors and the body worlds exhibits. *Canadian Journal of Science, Mathematics and Technology Education*, *18*(2), 98–113. 10.1007/s42330-018-0014-3.

[CR51] Pérez, D. G., Montoro, I. F., Alís, J. C., Cachapuz, A., & Praia, J. (2001). Para uma imagem não deformada do trabalho científico. *Ciência & Educação*, *7*(2), 125–153.

[CR52] Proctor, N. (2011). The Google art project: A new generation of museums on the web? *Curator: The Museum Journal*, *54*(2), 215–221. 10.1111/j.2151-6952.2011.00083.x.

[CR53] Rodrigues, N. (2001). Education: From human training to the construction of ethical subjects. *Educação & Sociedade*, *22*(76), 232–257. 10.1590/s0101-73302001000300013.

[CR54] Romanowski, J. P., & Ens, R. T. (2006). As Pesquisas Denominadas Do Tipo “Estado Da Arte” Em Educação. *Revista Diálogo Educacional*, *6*(19), 37–50.

[CR55] Sacramento, I., Monari, A. C. P., & Chen, X. (2020). The bat virus: Fake news and stereotyping of Chinese eating habits in the context of Covid-19. *Comunicação e Inovação*, *21*(47), 82–98.

[CR56] Santos, L. F. F., Pedrosa, L. L., & Aires, J. A. (2017). Contribuições da educação não formal para educação formal: um estudo de visitas de alunos da educação básica ao departamento de química da UFPR. *ACTIO: Docência Em Ciências*, *2*(1), 456-473. 10.3895/actio.v2n1.6804.

[CR57] Santos, M. C. T. M. (2001). Museu e educação: conceitos e métodos. *Ciências & Letras*, *31*, 307–323.

[CR58] Schultz, T. (2000). Massa media and the concept of interactivity: An exploratory study of online forums and reader email. *Media, Culture & Society*, *22*(2), 205–221. 10.1177/016344300022002005.

[CR59] Science Centre World Congress. (2008). *The Toronto declaration*. https://www.canadiansciencecentres.ca/resources/Documents/5WSC_Declaration_large.pdf. Accessed 25 Mar 2022.

[CR60] Shimizu, N. R. (2020). *Meaning relations about vaccination on Facebook*. [Master’s thesis, State University of Campinas]. Campinas, Base Acervus. 10.47749/T/UNICAMP.2020.1128900.

[CR61] Sinha, D. (2021). Hunger and food security in the times of Covid-19. *Journal of Social and Economic Development*, *23*(Suppl 2), 320–333. 10.1007/s40847-020-00124-y. 34720482 10.1007/s40847-020-00124-y PMC7883947

[CR62] Sisario, B. (2022). *Spotify and Joe Rogan Respond to Complaints About Covid Misinformation*. https://www.nytimes.com/2022/01/30/arts/music/spotify-joe-rogan-ceo.html. Accessed 13 Sep 2022.

[CR63] Sjöström, J., & Eilks, I. (2018). Reconsidering different visions of scientific literacy and science education based on the concept of Bildung. In Y. J. Dori et al. (Eds.), *Cognition, metacognition, and culture in STEM education: Learning, teaching and assessment*. Cham: Springer.

[CR64] Smithsonian National Zoological Park. (2020). *Field in focus: predicting pandemics*. https://artsandculture.google.com/story/LAVRHUr8BWm_Ow. Accessed 13 Sep 2022.

[CR65] Sotério, C. (2022). The provisional nature of science evidenced in times of pandemic. *Alternautas, 8*(1), 82-99, 10.31273/alternautas.v8i1.1121.

[CR66] Spotify. (2020). *How social distancing has shifted Spotify streaming*. https://newsroom.spotify.com/2020-03-30/how-social-distancing-has-shifted-spotify-streaming/. Accessed 13 Sep 2022.

[CR67] Taylor, E. W., & Neill, A. C. (2008). Museum education: A nonformal education perspective. *Journal of Museum Education*, *33*(1), 23–32. 10.1080/10598650.2008.11510584.

[CR68] Tuten, H., & Temesvari, L. (2013). Popular science journalism? Facilitating learning through peer review and communication of science news. *Journal of College Science Teaching*, *42*(4), 46–49.

[CR69] Vadja, A. (2020). Museums and online spaces. The society-building role of the museums during the pandemic. *Acta Universitatis Sapientiae Communicatio*, *7*, 72–53. 10.2478/auscom-2020-0004.

[CR70] van Leeuwen, T. (2015). Multimodality. In D. Tanen, H. Hamilton, & D. Schiffrin (Eds.), *The handbook of discourse analysis*. Malden/Oxford: Wiley Blackwell.

[CR71] Vayanou, M., Chrysanthi, A., Katifori, A., & Antoniou, A. (2020). Cultural heritage and social experiences in the times of COVID 19. *CEUR Workshop Proceedings*, *2687*, 2–5.

[CR72] Verde, A., & Valero, J. M. (2021). Virtual museums and Google arts & culture: Alternatives to the face-to-face visit to experience art. *International Journal of Education and Research*, *9*(2), 43–54.

[CR73] Vieira, C. L. (2007). *Pequeno manual de divulgação científica*, (3rd ed., ). Rio de Janeiro: Instituto Ciência Hoje.

[CR74] World Health Organization. (2020). *An ad hoc WHO technical consultation managing the Covid-19 infodemic: call for action*. https://apps.who.int/iris/bitstream/handle/10665/334287/9789240010314-eng.pdf. Accessed 25 Mar 2022.

[CR75] Zabala, A. (1995). *La práctica educativa: cómo enseñar*. Barcelona: Editorial Graó.

[CR76] Zabala, M. E., & Roura Galtés, I. (2006). Reflexiones teóricas sobre patrimonio, educación y museos. *Revista de Teoría y Didáctica de Las Ciencias Sociales*, *11*, 233–261 https://www.redalyc.org/articulo.oa?id=65201111.

[CR77] Zulli, D. (2018). Capitalizing on the look: Insights into the glance, attention economy, and Instagram. *Critical Studies in Media Communication*, *35*(2), 137–150. 10.1080/15295036.2017.1394582.

